# Circulating proliferative factors versus portal inflow redistribution: mechanistic insights of ALPPS-derived rapid liver regeneration

**DOI:** 10.3389/fonc.2024.1429564

**Published:** 2025-01-07

**Authors:** Shiran Zhang, Yu Ma, Xue Chen, Shuai Wu, Geng Chen

**Affiliations:** Department of Hepatobiliary Surgery, Daping Hospital, Army Medical University, Chongqing, China

**Keywords:** proliferative factors, portal hemodynamics, ALPPS, future liver remnant, liver regeneration

## Abstract

**Background:**

Associating liver partition and portal vein ligation for staged hepatectomy (ALPPS) can induce accelerated regeneration of future liver remnant (FLR) and effectively reduce the occurrence of liver failure due to insufficient FLR after hepatectomy, thereby increasing the probability of radical resection for previously inoperable patients with liver cancer. However, the exact mechanism by which ALPPS accelerates liver regeneration remains elusive.

**Methods:**

A review of the literature was performed utilizing MEDLINE/PubMed and Web of Science databases in March of 2024. The key words “liver regeneration/hypertrophy”, “portal vein ligation/embolization”, “two-stage hepatectomy”, “liver partition/split” and “future liver remnant” in combination with “mechanisms”, “hemodynamics”, “cytokines”, “growth factors” or “collaterals” were searched in the title and/or abstract. The references of relevant articles were reviewed to identify additional eligible publications.

**Results:**

Previously, a widely accepted view is that the primary role of liver splitting in ALPPS stage 1 is to accelerate liver regeneration by promoting proliferative factor release, but increasing evidence in recent years reveal that not the circulating factors, but the portal hemodynamic alternations caused by liver parenchyma transection play a pivotal role in ALPPS-associated rapid liver hypertrophy.

**Conclusion:**

Parenchyma transection-induced portal hemodynamic alternations are the main triggers or driving forces of accelerated liver regeneration following ALPPS. The release of circulating proliferative factors seems to be a secondary response to liver splitting and plays an auxiliary role in this process.

## 1 Introduction

Hepatectomy is the most important radical treatment for advanced liver cancer. Among newly diagnosed liver cancer patients, only 15-25% undergo surgical resection. A considerable number of patients are forced to forgo surgery due to the small size of the future liver remnant (FLR). Portal vein embolization (PVE) is an invaluable method for promoting liver regeneration, but the hypertrophy rate (10-69%) and waiting time (2-8 weeks) following PVE are highly variable (1). A two-stage hepatectomy procedure called associating liver partition and portal vein ligation for staged hepatectomy (ALPPS), which has emerged in recent years, can induce hypertrophy of the FLR in a much shorter time than PVE and provides more opportunities of radical resection for previously inoperable liver cancer patients. This technique constitutes a breakthrough in liver surgery and has attracted increased interest worldwide (2). Since ALPPS is very effective in promoting liver hypertrophy, an in-depth understanding of its mechanism of action is highly important for further optimization and modification. Early studies suggested that the redistribution of portal blood flow caused by portal vein ligation (PVL) and the release of circulating proliferative factors induced by liver parenchyma transection are two essential factors for ALPPS-derived FLR hypertrophy (3). However, in recent years, new findings from clinical and experimental studies have raised new doubts, controversies, and questions about the traditional paradigm of accelerated liver regeneration induced by ALPPS.

In this review, we summarized new findings and new theoretical developments on the mechanism of ALPPS-derived liver regeneration, discussed some controversial issues and new understandings, and described the trends and prospects of ALPPS for future research and clinical practice.

## 2 Methods

We performed a systematic literature search of the MEDLINE/PubMed, EMBASE and Web of Science databases in February 2024, using the key words “liver regeneration/hypertrophy”, “portal vein ligation/embolization”, “two-stage hepatectomy”, “liver partition/split” and “future liver remnant” in combination with “mechanisms”, “hemodynamics”, “cytokines”, “growth factors” or “collaterals”. All articles published in English and in peer-reviewed journals, or in print or online books over the last 15 years were included. We also checked the reference lists of each relevant study that resulted from this search for further appropriate articles. Older articles and research articles from other fields were also cited where appropriate to support the findings or statements.

## 3 Injury-induced circulating proliferative factors

### 3.1 Gene expression profile and signaling networks underlying ALPPS-induced accelerated regeneration

In 2014, Schlegel et al. (3) of the University of Zürich reported in a mouse model that extrahepatic organ ablation and ALPPS plasma injection could achieve FLR hypertrophy similar to that of ALPPS mice. The authors concluded that circulating factors in combination with PVL seemed to mediate enhanced liver regeneration in ALPPS, and the release of soluble mediators induced by injuries was not liver specific. Despite our increasing knowledge of gene expression patterns in normal and accelerated liver regeneration, comprehensive whole-genome analyses are required for full recognition of the underlying key regulators and pathways involved. Using RNA sequencing technology, Colak et al. (4) conducted a comprehensive analysis of the upstream regulatory factors and signaling pathways involved in the early postoperative period in rats subjected to ALPPS, partial hepatectomy (PH), and PVL. Cell cycle-related genes, transcription factors, DNA replication regulators, and cytokines were upregulated in all three groups in the early postoperative stage. Cluster analysis suggested that ALPPS and PH were associated with similar expression patterns of regulated genes that were significantly different from those associated with PVL. Borger et al. (5) quantitatively measured the activation of the intracellular signaling pathway (ISP) in liver tissue obtained from a mouse model of ALPPS, PH, and PVL based on whole-genome expression data. The activity of signaling pathways such as insulin-like growth factor 1 receptor (IGF1R), integrin-linked kinase (ILK), and interleukin 10 (IL-10) was enhanced after the first stage of ALPPS, while the activity of the interferon signaling pathway was reduced. Consistent with the finding that the gene expression profile after ALPPS showed more similar expression pattern to the PH than the PVL at the early phase of the regeneration, ALPPS shared nearly all the significantly affected ISPs at different postoperative time points with PH. The above results strongly suggest that the accelerated liver regeneration induced by ALPPS is more similar to that after hepatectomy.

### 3.2 Circulating proliferative factors involved in ALPPS-associated liver regeneration

#### 3.2.1 Inflammatory cytokines and growth factors

Dhar et al. (6) detected 29 cytokines in rat liver tissues by protein microarray and found that the expression levels of interleukin 6 (IL-6), cytokine-induced neutrophil chemoattractant-1 (CINC-1), interleukin 12 (IL-2), interleukin 13 (IL-13), and macrophage inflammatory protein-1α (MIP-1α) were significantly increased after the first stage of ALPPS. By serial analysis, Sparrelid et al. (7) reported that the serum hepatocyte growth factor (HGF) level was significantly elevated and positively correlated with the degree of FLR hypertrophy early after stage 1 in the ALPPS procedure. In a rat ALPPS model, Garcia-Perez et al. (8) reported that in addition to the significantly increased levels of proliferation-promoting cytokines such as IL-6, HGF, and tumor necrosis factor-α (TNF-α) after the first stage of ALPPS, the levels of antiproliferative cytokines such as IL-1β and transforming growth factor β (TGF-β) also significantly increased, suggesting that the proliferative response induced by ALPPS may be a complex process resulting from the interaction between proproliferative factors and antiproliferative factors (**Table 1**). It seems that these pro-inflammatory factors don’t affect tumor growth dynamics because several previous studies have showed that ALPPS did not result in tumor growth of colorectal liver metastases (CRLM) and hepatocellular carcinoma (HCC) (9, 10).

**Table 1:** The circulating proliferating factors involved in ALPPS-associated liver regeneration.

Category	Circulating factors	Influence on liver regeneration	References
Cytokines	TNF-α	TNF-α is a proinflammatory cytokine working via two distinct receptors, TNF receptor1 (TNFR1) and 2 (TNFR1) by the NF-κB signaling pathway.	(8)
	IL-2	IL-2 can activate and proliferate immune cells, and directly promote the proliferation of hepatocytes by activating signaling pathways associated with cell growth and division.	(6)
	IL-6	IL-6 is a cytokine playing active roles in liver regeneration. Signals are mediated via the AK-STAT pathway and the Ras-MAPK pathway.	(6–8)
	IL-1β	IL-1β stimulates the production of growth factors and cytokines that are important for liver regeneration, including HGF, TGF-β, and TNF-α.	(8)
	IL-10	IL-10 plays a crucial role in liver regeneration by suppressing inflammation, promoting hepatocyte proliferation, and regulating extracellular matrix remodeling.	(5)
	IL-12	IL-12 acts to initiate the inflammatory response necessary for liver regeneration, its excessive or prolonged activation can lead to deleterious effects such as fibrosis and chronic inflammation.	(6)
	IL-13	IL-13 has been shown to suppress the inflammatory response by inhibiting the production of inflammatory cytokines and chemokines. This anti-inflammatory action can help create a favorable environment for liver regeneration	(6)
	CINC-1	CINC-1 plays a pivotal role in liver regeneration by recruiting neutrophils, modulating the inflammatory response, and promoting hepatocyte proliferation and angiogenesis.	(6)
	MIP-1α	MIP-1α functions as a chemoattractant, guiding immune cells such as macrophages and neutrophils to the injured liver tissue.	(6)
Growth factors	HGF	HGF is a mitogen produced by mesenchymal cells and implicated in cell proliferation and angiogenesis via tyrosine phosphorylation of its receptor c-Met.	(7)
	FGF15	FGF15 serves as an important modulator of the regenerative process through binding to the FGF receptor 4 (FGFR4) and activating intracellular signaling cascades. ALPPS can trigger liver regeneration via intestinal Fxr-Fgf15 signaling pathway.	(16)
	TGF-β	Induction of apoptosis to correct excessive liver mass.	(8)
	TGF-α	TGF-alpha is a potent mitogenic factor that stimulates the proliferation of hepatocytes. It binds to the EGF receptor (EGFR) and activates intracellular signaling cascades that promote cell division and growth.	(8)
Circular RNAs	circ-0067724	circ-0067724 and circ-0016213 may act as miRNA sponges to regulating the expression of downstream target genes of miRNAs.	(18)
	circ-0016213		(18)
Other circulating factors	IHH	Early JNK1 activity induces IHH release from stellate cells. IHH promotes GLI1-CCND1 in hepatocytes to accelerate liver regeneration.	(11)
	Reg3α	Growing evidence links Reg3α proteins to regeneration of exocrine and endocrine tissues. The beneficial effect of Reg3α on acute liver failure was reported in mice.	(13)
	Reg3β	Reg3β induced in the ALPPS group activated the JAK2/STAT3 pathway and resulted in rapid liver regeneration.	(13)
	Bile acids	A potent FXR agonist (obeticholic acid, OCA) can accelerate liver regeneration after PVE in a rabbit model.	(15)

#### 3.2.2 Indian hedgehog

Langiewicz et al. (11) found that IHH was specifically and highly expressed in the early stage after ALPPS through gene chip screening. IHH is a secreted Hedgehog ligand produced by hepatic stellate cells through paracrine signaling, and its serum concentration peaks at 4 h after the first stage of ALPPS. Moreover, the downstream genes of the Hedgehog signaling pathway are also activated. After the injection of recombinant IHH into PVL mice, liver regeneration similar to that of ALPPS can be simulated. After the injection of an IHH-neutralizing antibody into ALPPS mice, FLR hypertrophy was significantly inhibited. Further research (12) has shown that c-June N-terminal kinase 1 (JNK1) is an upstream regulatory molecule of IHH. Blocking JNK1 before ALPPS can inhibit hepatocyte hypertrophy, which is accompanied by downregulation of the activity of the IHH–GLI1–CCND1 axis. Administration of recombinant IHH restored ALPPS-like liver regeneration and upregulated JNK activity. This study suggested that JNK1-mediated paracrine signaling in IHH is necessary for the acceleration of liver regeneration, and that the JNK1–IHH axis may be a unique mechanism by which ALPPS promotes liver regeneration.

#### 3.2.3 Reg3α and Reg3β

Recently, Otsuka et al. (13) reported that the JAK2/STAT3 signaling pathway may play a key role in ALPPS-induced liver regeneration. After blocking this signaling pathway with the JAK2-specific inhibitor G490, the weight of the right median lobe (RML) to body weight (RML/BW) ratio did not change significantly in the PVL group, while the RML/BW ratio was significantly lower in the ALPPS group. Immunohistochemistry showed strong staining for phosphorylated signal transducer and activator of transcription 3 (p-STAT3) in the nuclei of hepatocytes in the ALPPS group, while no p-STAT3 staining was found in the hepatocytes of the PVL group. They further analyzed more than 20,000 genes using a cRNA microarray and found that regenerating islet-derived 3α (Reg3α) and Reg3β were upstream factors that activate the Janus-activated kinase 2 (JAK2)/STAT3 signaling pathway after ALPPS.

#### 3.2.4 Bile acids

The significance of bile acid for liver regeneration after PH and partial liver transplantation (PLT) has been well studied at both the animal model and clinical levels. The bile salt-activated transcription factor farnesoid X receptor (FXR) is a key mediator of proliferative bile salt signaling, and is assumed to play a role in the early phase of compensatory liver regeneration (14). Olthof et al. (15) reported that obeticholic acid (OCA), a potent FXR agonist, accelerated liver regeneration after PVE in a rabbit model. Daradics et al. (16) reported that ALPPS induced more extensive elevation of systemic and portal bile acid levels (p<0.05) than PVL in a rat model. Bile acid-activated mitotic signals in ALPPS could be characterized by the activation of the intestinal Fxr pathway rather than hepatic Fxr signaling.

### 3.3 The underlying role of circulating proliferative factors in ALPPS

Liver partition can accelerate liver regeneration only when combined with PVL, which suggests that the redistribution of portal blood flow is the physiological basis for ALPPS-induced liver regeneration. The exact role of circulating factors in accelerated regeneration, either as a passive followers or active participants, is still controversial and needs to be elucidated. Transcriptome studies revealed that ALPPS and PH shared many significantly regulated genes whose expression were not otherwise significantly changed in PVL. The post-ALPPS/-PH alterations in gene expression during liver regeneration were mostly due to the time effect, not the operation type (4, 5). Whether the different spatiotemporal expression patterns of regenerative genes are the main cause of the unique liver regeneration induced by ALPPS is still inconclusive (17–19). In an experimental study conducted by Otsuka et al. (13), a rat model with a liver split inside the portal vein ligated lobe (PiLL) was created. According to Schlegel’s theory (3), if increased inflammatory cytokines due to liver partition are responsible for the accelerated liver regeneration induced by ALPPS, rapid liver hypertrophy should be achieved regardless of where the liver is split. However, FLR hypertrophy was significantly greater in the ALPPS group than in the PiLL group, whereas the levels of cytokines/growth factors, including IL-6, TNF-α, and HGF, were comparable between the two groups. It is obvious that an increase in cytokines/growth factors was not enough to describe the mechanism of rapid liver hypertrophy in ALPPS. Recently, Masuo et al. (20) reported that the serum concentrations of IL-6 and TNF-α, in the short term (1, 4, and 6 h) after surgery did not differ between the ALPPS and PVL groups, although the ALPPS group showed a greater increase in the FLR and a higher Ki-67 labeling index than did the PVL group. Additionally, suppression of inflammatory cytokines using GdCl_3_ did not suppress liver regeneration. These results suggest that the induction of circulating proliferative factors in the early phase after ALPPS is not necessarily a major factor in accelerating liver regeneration.

## 4 Portal hemodynamic alternations induced by liver partition

### 4.1 The impact of liver partition on portal hemodynamics

Most of the early studies attributed the changes in hepatic hemodynamics observed during ALPPS to portal vein ligation, ignoring the impact of hepatic parenchyma transection on hepatic hemodynamics. In a clinical study, Chan et al. (21) reported that after right portal vein ligation, the portal vein blood flow in the FLR increased from 76.6 ml/100 gm/min to 193.7 ml/100 gm/min, and further increased to 259 ml/100 gm/min after hepatic parenchymal transection. In a rat PVL model (16), liver transection further increased portal pressure in the non-ligated right median lobe and further decreased the microcirculatory flow in the ligated left median lobe. Our recent study (22) showed that complete liver splitting along the demarcation line induced higher portal blood velocity, blood flow and portal pressure than partial or ectopic liver splitting following PVL. These results revealed the significant impact of liver partition on portal hemodynamics in the reserved liver.

### 4.2 Intrahepatic porto-portal collaterals

The formation of intrahepatic PPCs has been described previously in patients with portal vein thrombosis. Denys et al. (23) reported the first case with collaterals connecting segment 4 and segment 8 portal vein system arising from the left hemiliver, with the right hemiliver, who failed to induce hypertrophy after ligation of the right portal vein. Because the intrahepatic venous system is naturally anastomotic, such veins can easily hypertrophy. Proximal occlusion of the portal vein (PVL) allows distal collateral reentry of portal flow through more distal branches, resulting in a progressive intrahepatic cavernoma (24, 25). The authors concluded that filling of the portal venous system through such collaterals may have hindered hypertrophy in this patient. They also pointed out the development of such collaterals illustrates one of the limitations of PVL when compared with PVE. Van Lienden et al. (26) performed CT scans and intraoperative portography in 18 patients after PVE or PVL. They demonstrated intrahepatic PPCs in all PVL patients and some PVE patients. The collaterals could be demonstrated as 1-2 mm large vessels connecting larger portal vein branches between segment 4 and segments 5 and 8. Wilms et al. (27) compared a mini-pig model of PVL with PVE and demonstrated that PVL induced PPCs that allowed orthograde flow into the entire liver in *ex situ* angiograms 7 days after surgery. Because PVE is more effective to increase the FLR, the authors concluded that the formation of collaterals between occluded and nonoccluded liver parts may be the cause of inferior regeneration in the ligation group. Deal et al. (28) reported, in a porcine model, that both the PVL group and the partial ALPPS group formed substantial new portal vein collaterals to varying degrees on postoperative day 7, and the number of collaterals was inversely proportional to the FLR growth rate. Our previous study (29) showed that in some patients after the first stage of ALPPS, contrast-enhanced ultrasound or enhanced computed tomography (CT) revealed blood flow in the distal branches of the ligated portal vein, indicating the presence of arterio-portal shunts (APSs) and PPCs. By using rescue radiofrequency ablation (RFA)/percutaneous ethanol injection (PEI) to obliterate the APSs and PPCs, the kinetic growth rate (KGR) of FLR increased significantly to 4% compared with that before the rescue procedures (1.5%, P<0.05). Therefore, current evidence clearly suggests that hypertrophy after ALPPS is likely more rapid due to the cutoff of collaterals by parenchyma transection, which may also be the cause of the further increase of FLR portal pressure after liver split (30).

### 4.3 Shear stress and nitric oxide release

Shear stress is directly caused by blood flow and exerts shear forces on liver sinusoid endothelial cells (LSECs) and adjacent hepatic stellate cells (HSCs) in hepatic sinusoids. Endothelial nitric oxide synthase (eNOS) activation, followed by nitric oxide (NO) induction, has been reported to promote liver regeneration in response to partial hepatectomy (31). When endothelial cells are stimulated by shear stress or vascular endothelial growth factor (VEGF), phosphoinositide 3-kinase (PI3K) is activated and phosphatidylinositol 3,4,5-trisphosphate (PIP3) is produced, which activates the PI3K-Akt pathway and activates downstream signals such as eNOS (32). Masuo et al. (19) reported that phospho-Akt Ser^473^ and phospho-eNOS Ser^1177^ levels were greater in the ALPPS group than in the PVL group, suggesting that activation of the Akt-eNOS pathway contributes to accelerated liver regeneration in ALPPS. Although the exact shear stress within hepatic sinusoids or the space of Disse *in vivo* has not been measured directly in human or animal models so far due to the tiny scale and varied sizes of the hepatic sinusoids as well as the vascular permeability induced by LSEC fenestrae, the increases of portal blood flow and pressure in the remnant liver following ALPPS stage-1, are expected to increase shear stress and induce NO production, which will promote rapid liver hypertrophy.

### 4.4 Hepatic artery buffering response and liver hypoxia

Changes in portal inflow also have a significant impact on hepatic arterial blood flow. In 1981, Lautt et al. (33) firstly proposed the concept of the hepatic arterial buffer response (HABR). This unique mechanism represents the ability of the hepatic artery to produce compensatory flow changes in response to changes in portal venous flow; i.e., if portal blood flow is reduced, the hepatic artery dilates, and the hepatic artery constricts, if portal flow is increased. The HABR can also be observed after the first stage of ALPPS (34). Due to ligation of the tumor-side portal vein, the blood flow in the reserved side portal vein markedly increased, resulting in a decrease in the blood flow in the reserved side hepatic artery to maintain constant total sinusoidal blood flow in the FLR.

Schadde et al. (35) reported that the artery blood flow of the FLR was reduced by nearly 40% after the first stage of ALPPS in a rat model, which directly led to a hypoxic state in the growing liver. To further clarify the effect of hypoxia on the regeneration of the FLR, they administered the prolyl hydroxylase inhibitor dimethyloxalylglycine (DMOG) to specifically activate hypoxia-related signaling pathways in PVL rats and found that FLR hypertrophy was significantly accelerated. At 24 h, an increase of 60% ± 14% was observed, and at 72 h, an increase of 134% ± 21% was noted. Myoinositol trispyrophosphate (ITPP) is an allosteric effector that can reduce the oxygen affinity of hemoglobin and promote the release of oxygen from red blood cells. ITPP can inhibit the proliferation of tumors by blocking their hypoxic state. In this study, treatment of ALPPS rats with ITPP reduced FLR hypertrophy to a level comparable to that in the PVL group. Most interestingly, the local application of DMOG on the surface of the liver can promote the proliferation of hepatocytes. Ki67 staining revealed a hypertrophic zone with a thickness of approximately 1 mm on the liver surface. The above evidence suggested that hypoxia may be one of the major accelerators of ALPPS-derived liver regeneration.

### 4.5 Role of parenchyma transection-induced portal hemodynamic alternations in accelerated liver regeneration

**Figure 1:**
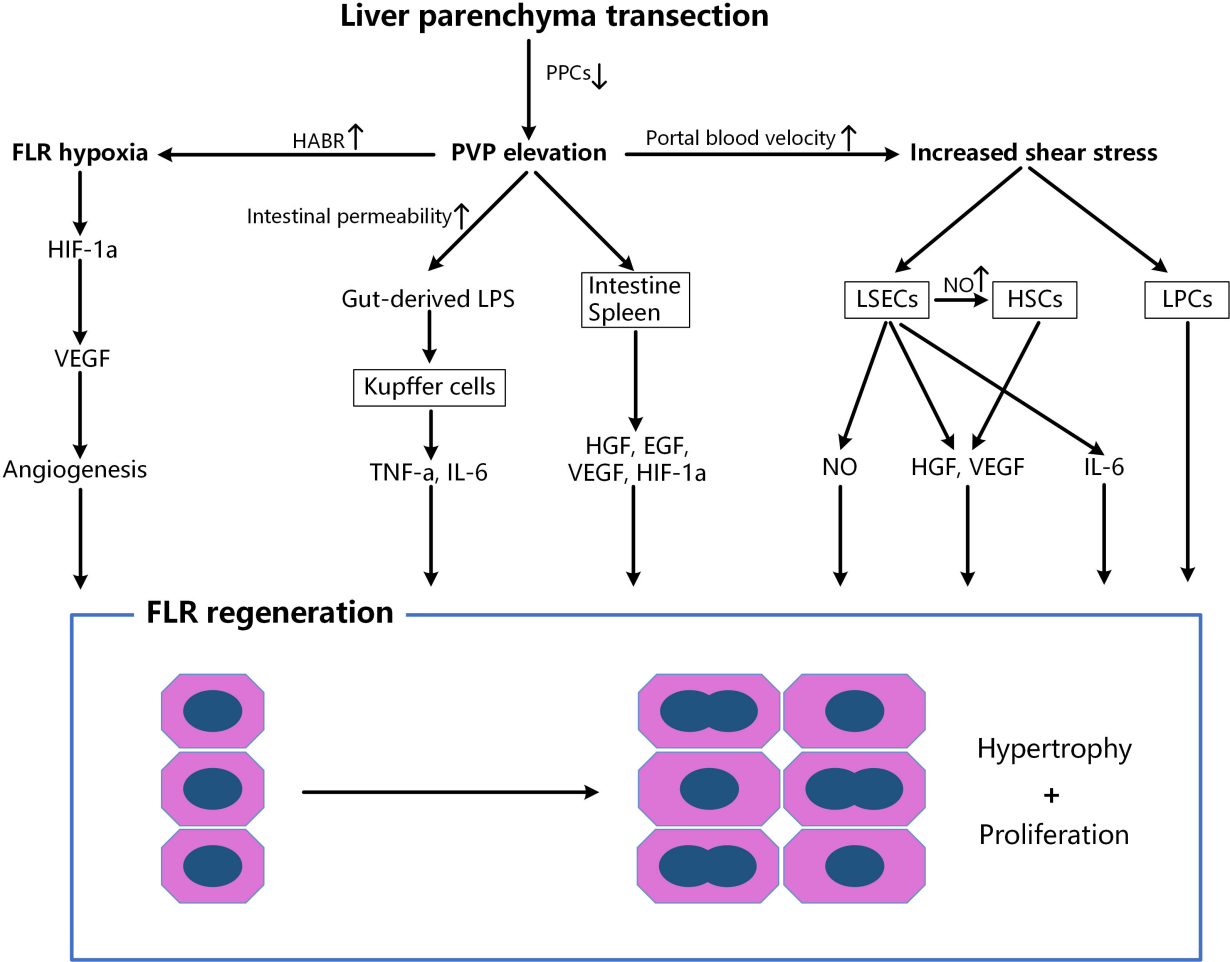
Factors associated with accelerated liver regeneration after liver parenchyma transection. After splitting, the cutting off of PPCs further increases the portal pressure, portal inflow in the reserved lobe and hepatic artery flow in the ligated lobe, which leads to the enhanced FLR hypoxia and shear stress. FLR, future liver remnant; PPCs, porto-portal collaterals; HABR, hepatic arterial buffer response; EGF, epidermal growth factor; HGF, hepatocyte growth factor; HIF-1α, hypoxia inducible factor 1-alpha; HSCs, hepatic stellate cells; IL, interleukin; LSECs, liver sinusoidal endothelial cells; LPCs, liver progenitor cells; LPS, lipopolysaccharide; NO, nitric oxide; PVP, portal vein pressure; TNF, tumor necrosis factor; VEGF, vascular endothelial growth factor.

According to the paradigm of “portal inflow redistribution + circulating proliferative factors”, liver parenchymal transection does not seem to be indispensable for accelerated FLR hypertrophy (3). Therefore, many improvements for the first-stage ALPPS procedure have been made during the past 10 years to minimize complications during the interstage phase and improve outcomes after the second stage, for example, partial liver partition (36), radiofrequency-assisted liver partition (37), and round-the-liver tourniquet have been used to replace complete liver split (38–40), aiming to reduce parenchymal damage, bile leak and manipulation of the hepatic hilum. However, some problems have gradually emerged in clinical practice. We observed in patients (41) that the interstage interval for modified ALPPS without liver parenchymal transection was significantly longer than that for classical ALPPS (18-22 d vs. 7-12 d, P<0.05). A clinical study from the University of Hong Kong (21) showed that the split completeness of the hepatic parenchyma was closely related to the degree of FLR hypertrophy following PVL. The waiting time for FLR hypertrophy was significantly shorter (7 d vs. 10.5 d, P<0.05) and the rate of FLR hypertrophy was greater (11.5 ml/d vs. 5.5 ml/d P<0.05) in the complete liver split group than in the partial split group. In a porcine ALPPS model (28), the FLR increase rate in the complete transection group was twice that in the partial transection group (64% vs. 32%, P<0.05). It is interesting that the serum aminotransferase levels of the complete and partial liver split groups were comparable after the stage-1 ALPPS procedure. Recently, we found (22) that a liver split along the demarcation line in a rat model could further increase the portal pressure of the FLR, which is associated with accelerated liver hypertrophy. No significant differences in terms of biochemical or pathological indicators reflecting liver injury (e.g., aminotransferase, bilirubin, necrosis scores), or serum cytokine levels, were detected among the groups with the same extent of liver split. Moreover, a recent observational study revealed that FLR hypertrophy depends on initial FLR volume and a smaller estimated FLR is associated with greater degree of hypertrophy, mainly due to the higher portal pressure and portal flow per gram of the smaller FLR (42). In summary, these results strongly suggest that liver parenchyma transection is not optional, but is essential for ALPPS. It seemed that the accelerated regeneration induced by liver splitting was mainly achieved by the transection of PPCs and subsequent portal hemodynamic alternations of the FLR (**Figure 1**).

Compared with liver regeneration after a viral- or drug-induced liver injury, that of post-PH has several distinct features, such as hemodynamic changes in portal venous flow or pressure, and tissue ischemia/hypoxia (43). Because ALPPS shares nearly all the significantly affected genes and ISPs with PH (4, 5), the stage-1 procedure of ALPPS can be regarded as either an “incomplete PH” or an “enhanced PVL”. From this point of view, we believe that parenchyma transection-induced portal hemodynamic alternations are the main triggers or driving forces of accelerated liver regeneration.

## 5 Current trends and future prospects of ALPPS

The clinical application of ALPPS worldwide has significantly increased the resection rate of liver malignancies, deepened our understanding of the mechanism of liver regeneration, and promoted the development of regenerative liver surgery (1). Increase in FLR after ALPPS stage-1 ranging from 65 to 110% and interval between the stage 1 and 2 procedures ranging from 6 to 15 days have been reported (1, 2). However, some problems still remain. The first is the surgical trauma. No matter what kind of method for liver partition is adopted, substantial trauma is inevitable, which has a great negative impact on patients physiologically and psychologically. The second problem is that the functional increase of liver remnant usually cannot catch up with the volume increase following the stage-1 ALPPS procedure (44). ALPPS-associated liver regeneration creates quantity, but not quality liver tissue, which leads to a high incidence of post-hepatectomy liver failure (PHLF) after the stage-2 ALPPS procedure (45), especially for the patients with liver cirrhosis/fibrosis. Lengthening the interstage interval to allow time for FLR maturation has been suggested, but at least partially mitigates against the benefits provided by the increased KGR.

**Figure 2:**
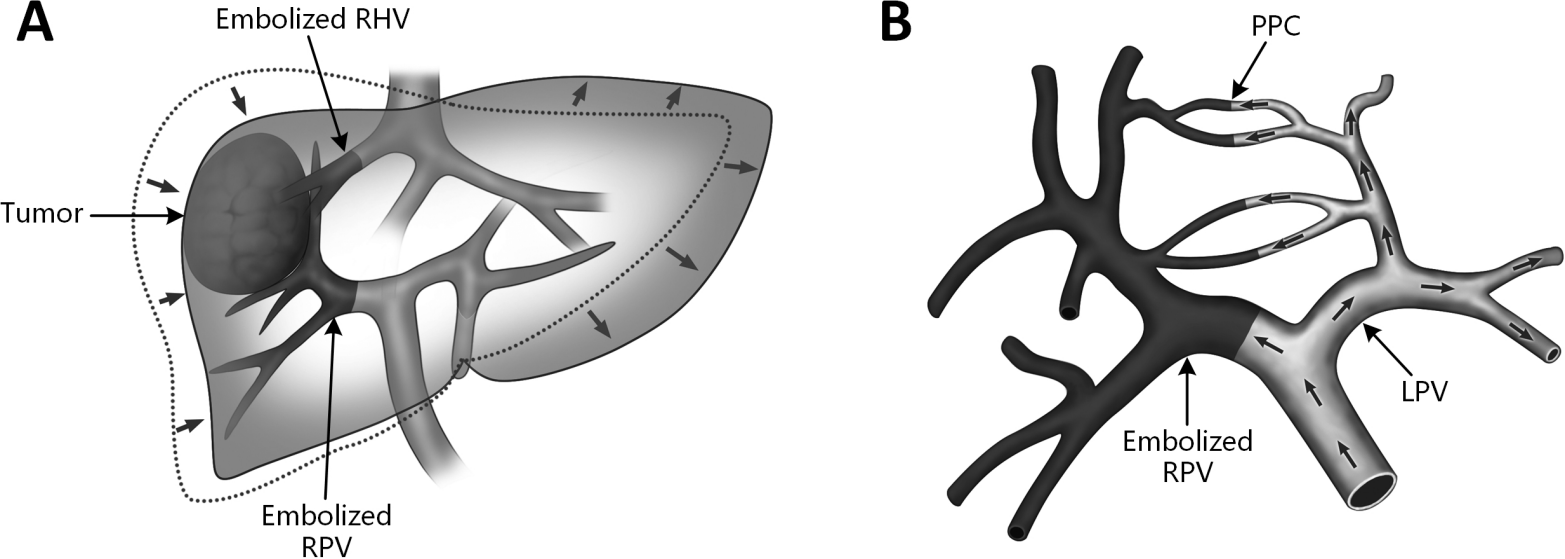
Schematic diagrams of two endovascular FLR augmentation techniques. **(A)** liver venous deprivation (LVD). Combination of right portal vein (RPV) embolization and right hepatic vein (RHV) embolization before major hepatectomy; **(B)** terminal branch portal vein embolization (TBPVE). By using n-butyl cyanoacrylate gel (NBCA) as the embolic material, smaller branches of the portal vein can be embolized, completely blocking the collateral circulation between the two hemilivers. PPC, porto-portal collateral; LPV, left portal vein; RPV, right portal vein, RHV, right hepatic vein.

Experimental studies have demonstrated that portal collateralization and neoangiogenesis are likely the result of portal hyperflow restricted by a limited venous outflow bed in the growing lobe. Therefore, it can be abrogated by occluding the venous outflow on the side of portal inflow occlusion (35–47). Guiu et al. (48) firstly reported the use of liver venous deprivation (LVD), i.e. simultaneous PVE and ipsilateral hepatic vein embolization (HVE) before hepatectomy to promote FLR hypertrophy (**Figure 2A**). The FLR increased by 45% after an average of 23 days. Furthermore, combination of right portal vein embolization and right and middle hepatic vein embolization (extended liver venous deprivation, eLVD) (49) can provide more rapid increase in FRL volume and function (53.7% and 64.3% at day 7, respectively). Another technique to disrupt naturally occurring PPCs is the terminal branch portal vein embolization (TBPVE), firstly reported by Peng et al. (50), by using a liquid embolic material, N-butyl-cyanoacrylate (NBCA). Theoretically, TBPVE can embolize smaller branches of the portal vein and completely block the collateral circulation between the two hemilivers (**Figure 2B**). For patients with primary liver cancer and cirrhosis, the rate of FLR hypertrophy at 14 days after TBPVE reached 52.1%, which was much greater than that after classic PVE. A randomized controlled trial (BestFLR trial) (51) compared the use of NBCA combined with ethiodized oil and standard polyvinyl alcohol (PVA) particles plus coils for preoperative PVE. Faster and superior liver hypertrophy was observed in the NBCA group than in the PVA group 14 days and 28 days after PVE (46% vs 30% and 57% vs 37% [P < 0.001], respectively). 87% of participants in the NBCA group received hepatectomy 14 days after PVE. The incidence of PHLF in the NBCA group was lower than that in the PVA group (13% vs. 27%).

These results strongly suggest that the efficacy of PVE in promoting liver hypertrophy can be greatly enhanced by technical improvements (e.g., adding ipsilateral hepatic vein embolization, using NBCA as embolic material). Current data show that the FLR growth rate following LVD or TBPVE has exceeded 50%, and the time interval between two stages has been reduced to 2-4 weeks, which are gradually getting close to those of ALPPS (52–54). Moreover, the discrepancy between volumetric growth and functional increase of the FLR is not observed in LVD and TBPVE (49, 55). Considering that PVE is much more widespread than ALPPS and that ALPPS has relatively higher rates of PHLF, morbidity, and mortality, the endovascular approach eliminates the necessity of two-stage surgery and is highly attractive to reduce the surgical severity of the ALPPS (56). It might be safer to perform TBPVE or LVD first in patients scheduled for major hepatectomy with insufficient FLR, only proceed to ALPPS when the hypertrophy cannot meet the need of liver resection. Future studies should focus on the applicability, safety, efficacy and long-term prognosis of the PVE-derived modifications through multi-center collaborations. A “step-up” strategy needs to be developed for the chosen of suitable FLR augmentation techniques in the clinical practice of regenerative liver surgery (57).

A comprehensive assessment of FLR function reserve and postoperative mortality risk is also essential for reducing the incidence of PHLF following ALPPS. Direct measurement of regional liver function through hepatobiliary scintigraphy or Gd-EOB-DTPA-enhanced magnetic resonance imaging (MRI) may be helpful to protect from liver failure after stage 2 (44, 58). The ALPPS risk score based on patient individual characteristics has been created in order to estimate and predict the 90-day or in-hospital mortality risk of patients either upfront before stage 1 or before stage-2 surgery, which provides an assisting tool for the hepatobiliary surgeon to guide treatment decisions (59). It should be noted that the hyaluronic acid (HA), a serum predictive marker for PHLF with high sensitivity and specificity, may have a potential role in ALPPS risk assessment (60, 61). A large prospective randomized cohort study is needed to elucidate predictive diagnostic value of perioperative HA levels for PHLF following ALPPS.
